# Epigenetic regulation of programmed cell death in hypoxia-induced pulmonary arterial hypertension

**DOI:** 10.3389/fimmu.2023.1206452

**Published:** 2023-09-11

**Authors:** Yuan Jiang, Shasha Song, Jingxin Liu, Liyuan Zhang, Xiaofei Guo, Jiayao Lu, Lie Li, Chao Yang, Qiang Fu, Bin Zeng

**Affiliations:** ^1^ College of Pharmacy, Harbin Medical University, Harbin, Heilongjiang, China; ^2^ College of Pharmacy, Shenzhen Technology University, Shenzhen, China; ^3^ Shanghai Baoxing Biological Equipment Engineering Co., Ltd, Shanghai, China; ^4^ National Engineering Research Center for Marine Aquaculture, Institute of Innovation & Application, Zhejiang Ocean University, Zhoushan, China; ^5^ Shenzhen Reyson Biotechnology Co., Ltd, Shenzhen, China; ^6^ Nanjing Evertop Electronics Ltd., Nanjing, China

**Keywords:** pulmonary arterial hypertension (PAH), apoptosis, autophagy, pyroptosis, ferroptosis, DNA methylation, histone modification, non-coding RNA (ncRNA)

## Abstract

Pulmonary arterial hypertension (PAH) is a severe progressive disease that may cause early right ventricular failure and eventual cardiac failure. The pathogenesis of PAH involves endothelial dysfunction, aberrant proliferation of pulmonary artery smooth muscle cells (PASMCs), and vascular fibrosis. Hypoxia has been shown to induce elevated secretion of vascular endothelial growth factor (VEGF), leading to the development of hypoxic PAH. However, the molecular mechanisms underlying hypoxic PAH remain incompletely understood. Programmed cell death (PCD) is a natural cell death and regulated by certain genes. Emerging evidence suggests that apoptotic resistance contributes to the development of PAH. Moreover, several novel types of PCD, such as autophagy, pyroptosis, and ferroptosis, have been reported to be involved in the development of PAH. Additionally, multiple diverse epigenetic mechanisms including RNA methylation, DNA methylation, histone modification, and the non-coding RNA molecule-mediated processes have been strongly linked to the development of PAH. These epigenetic modifications affect the expression of genes, which produce important changes in cellular biological processes, including PCD. Consequently, a better understanding of the PCD processes and epigenetic modification involved in PAH will provide novel, specific therapeutic strategies for diagnosis and treatment. In this review, we aim to discuss recent advances in epigenetic mechanisms and elucidate the role of epigenetic modifications in regulating PCD in hypoxia-induced PAH.

## 1 Introduction

Pulmonary arterial hypertension (PAH) is a fatal cardiovascular disease, also known as malignancy of the cardiovascular system. PAH is characterized by a progressive increase in pulmonary vascular resistance (PVR) and pulmonary vascular remodeling, leading to right ventricular remodeling and ultimately death from right ventricular failure (1, 2). Pulmonary vascular remodeling is a common pathological feature of PAH and encompasses multiple cell types within the blood vessel wall, including endothelial cells (ECs), pulmonary artery smooth muscle cells (PASMCs), fibroblasts, pericytes, and circulating inflammatory cells (3). Although the pathological mechanism of PAH remains incompletely understood, the basic pathological processes are related to the interplay among diverse cellular types in the pulmonary vascular wall, such as abnormal cell energy metabolism, cell differentiation, apoptosis resistance, excessive cell proliferation, and extracellular matrix deposition (4). Therefore, further understanding of cellular processes and mechanisms involved in PAH will provide more efficient therapeutic strategies.

Cell death mechanisms are generally classified into two distinct types: programmed cell death (PCD) and necrotic cell death. PCD is required to control the balance of normal cell homeostasis (5). The canonical form of PCD is apoptosis. Additionally, many other types of programmed cell death, including autophagy, pyroptosis, and ferroptosis, have been characterized (6). Nevertheless, the molecular mechanisms in different types of PCD are complex and usually provoke through a variety of independent pathways. Therefore, the discovery of the underlying mechanisms of PCD is urgently needed.

Epigenetics is indispensable for regulating gene expression, protein transcription, and translation in many biological processes, including DNA methylation, histone modification, non-coding RNA molecules, and *N* ^6^-methyladenosine methylation (7). Epigenetics differs from classical genetics, which is independent of changes in genomic DNA base sequence (8). Epigenetic modifications can be added to molecules by transferases, also known as “writers” and removed by “erasers”. Crucially, the molecular effects of epigenetics rely on the recognition by specific proteins, also known as “readers” (9). Previous studies have suggested an association between epigenetic modifications and various pathological processes (10). Recently, the underlying functional machinery of epigenetic modifications in PAH has also been attracting extensive attention.

In this review, we focus on the latest advances in epigenetic modifications, such as DNA methylation, histone modification, non-coding RNA molecules, and *N* ^6^-methyladenosine methylation related to PCD in hypoxia-induced PAH, thereby identifying the potential therapeutic strategy for PAH.

## 2 Programmed cell death in PAH

PAH is characterized by abnormal functioning of various cell types, including pulmonary arterial endothelial cells (PAECs), PASMCs, fibroblasts, and inflammatory cells (11). Studies have indicated that abnormal proliferation and anti-apoptotic phenotype of PAECs and PASMCs contribute to the occlusion of pulmonary arterioles, resulting in right heart hypertrophy and eventual cardiac failure. Additionally, fibroblasts isolated from the models of pulmonary hypertension exhibit a hyperproliferative, apoptosis-resistant, and proinflammatory phenotype (12). Chronic inflammation plays an essential role in PAH. Pulmonary vasculopathy has been identified with the presence of immune cell infiltrates, consisting of macrophages, lymphocytes, and mast cells. Autophagy plays an essential role in inflammasome activity. However, whether autophagy-mediated inhibition of inflammasome activity is involved in regulating the progression of PAH remains unclear (13). Furthermore, the presence of interleukin (IL)-1β, IL-18, and pyroptosis, which are end products of inflammasome activation, serves as a pivotal biomarker for PAH (14). These observed changes suggest a connection between programmed cell death mechanisms, including apoptosis, autophagy, pyroptosis, and even ferroptosis in the key cells associated with PAH.

### 2.1 Apoptosis

Apoptosis, the first identified form of programmed cell death, is a crucial process by which cells autonomously regulate their own death under physiological or pathological conditions (15). The initiation of apoptosis is dependent on morphological changes in cell structure and the activation of cysteine and aspartic protease processes (16). Mechanically, apoptosis is mainly activated by two pathways: the intrinsic pathway (the mitochondrial pathway of apoptosis) and the extrinsic pathway of apoptosis (the death receptor pathway of apoptosis) (17). The intrinsic apoptosis is dependent on factors released from the mitochondria and can be triggered by a series of external stimuli such as hypoxia, reactive oxygen species, and viruses. Conversely, extrinsic apoptosis is initiated by the specific death ligands binding to the death receptors (18).

**Figure 1:**
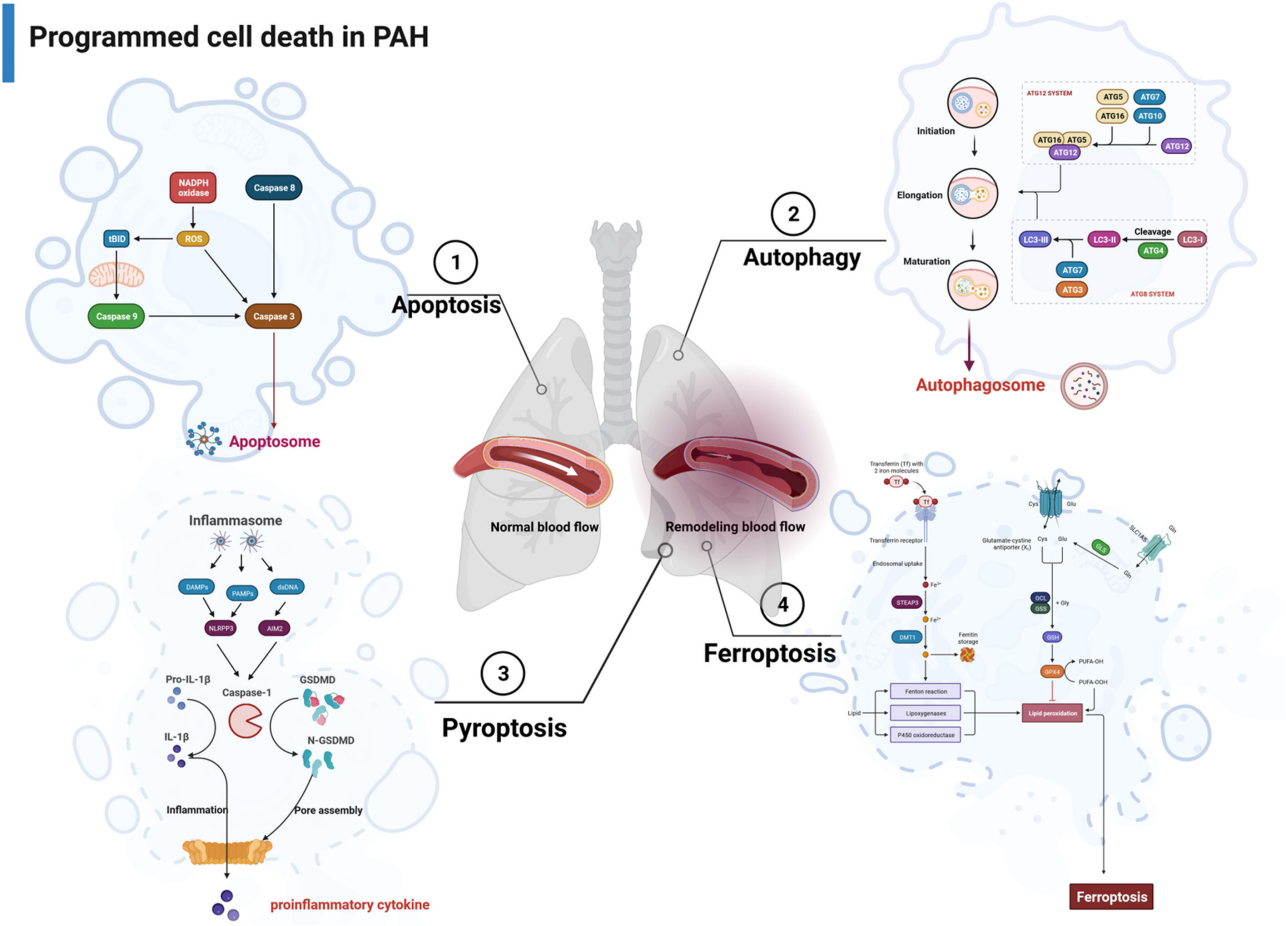
The major pathways associated with apoptosis, autophagy, pyroptosis, and ferroptosis in PAH. PAH, pulmonary arterial hypertension.

Multiple studies have suggested that apoptosis is associated with pulmonary vascular remodeling in PAH. Under physiological conditions, apoptosis plays a crucial role in maintaining organ and tissue integrity by regulating the balance between cell proliferation and programmed cell death (19). Nevertheless, the underlying molecular mechanisms of apoptosis in PAH remain to be explored. In a study by Chowdhury et al., it was discovered that dysfunctional bone morphogenetic protein receptor II (BMPRII) impairs apoptosis *via* the BMPRII-ALK1-Bcl-xL pathway in PAH (20). Wang et al. demonstrated that mutations in the bone morphogenetic protein 9 (BMP9) contribute to the etiology of PAH by impairing the anti-apoptotic abilities of PAECs (21). Additionally, Cao et al. reported that prohibitin 1 (PHB1) contributes to PAH by balancing PASMC proliferation and apoptosis, which involves AKT phosphorylation (22). In Jiang’s study, prostaglandin E1 (PGE1) modulates the apoptotic properties of mesenchymal stem cells (MSCs) by regulating the HIF pathway, thereby enhancing the therapeutic potential of the MSCs in PAH (23). He et al. conducted studies that revealed the inhibition of apoptosis in distal pulmonary artery smooth muscle cells (dPASMCs) by PRDC, which was able to reverse the effects of BMP2/4 on the upregulation of apoptosis-associated proteins such as caspase 3, caspase 9, and Bax while downregulating the expression of Bcl-2 (24). Novoyatleva et al. found that deficiency of Axl aggravated PAH and abrogated bone morphogenetic protein receptor 2 (BMPR2) signaling, thereby increasing pulmonary endothelial cell apoptosis (25). Recently, Ruffenach et al. reported that the RNA-binding protein HNRNPA2B1 (heterogeneous nuclear ribonucleoprotein A2B1; A2B1) silencing in PASMCs led to a decrease in proliferation and resistance to apoptosis, which is expected to become a therapeutic target for PAH (26) (**Figure 1**).

### 2.2 Autophagy

Autophagy, a form of programmed cell death, plays a pivotal role in the self-renewal process of eukaryotic cells (27). It involves the degradation of cytoplasmic proteins and damaged organelles through the action of lysosomes and is regulated by a set of autophagy-related genes (Atgs). Autophagy can be classified into three forms—macroautophagy, microautophagy, and chaperone-mediated autophagy (CMA)—among which macroautophagy is the most widely studied (28). The process of autophagy can be broken down into several successive steps: initiation and nucleation of autophagosome → elongation and formation of autophagosome → fusion with lysosomes (29).

Autophagy can be induced by cellular stress responses such as hypoxia and nutrient deficiency. Studies have confirmed that the level of autophagy is upregulated during the PAH, which plays an important role in vascular remodeling. Studies by Zhai et al. discovered that activation of AMPK prevents PAH by suppression of NF-κB-mediated autophagy activation (30). In another study, Chang et al. proposed Aldehyde Dehydrogenase 2 (ALDH2) protected against hypoxia-induced PASMC proliferation *via* inhibition of ERK1/2-mediated autophagy (31). Ning et al. found that β-arrestin1 inhibits hypoxia-induced autophagy *via* the Akt/mTOR signaling pathway (32). Moreover, Gomez-Puerto et al. observed an increase in levels of microtubule-associated protein 1 light chain 3 beta (MAP1LC3B) in PAH, while pulmonary microvascular endothelial cells (MVECs) from PAH patients exhibited heightened autophagic flux (33). Feng et al. further elucidated the promotion of PASMC proliferation and pulmonary vascular remodeling by high mobility group box-1 (HMGB1) through the activation of the ERK1/2/Drp1/Autophagy/BMPR2/Id1 axis (34). Jin et al. demonstrated that farnesyl diphosphate synthase (FDPS) contributes to active small G protein-induced autophagy during PAH (35). In a separate investigation, it was found that glucagon-like peptide-1 (GLP-1) receptor agonist, liraglutide, can suppress the proliferation of PASMCs by inhibiting cellular Drp1/nicotinamide adenine dinucleotide phosphate (NADPH) oxidase (NOX) pathways and Atg-5/Atg-7/Beclin-1/LC3β-dependent pathways of autophagy in PAH (36). He et al. conducted a study showing that quercetin enhances hypoxia-induced autophagy through the FOXO1-SENS3-mTOR-dependent pathway in PASMCs (37). In a systematic study, Yamanaka et al. showed that TP53-induced glycolysis and apoptosis regulator (TIGAR) regulates PASMC proliferation and migration by inhibiting autophagy and improving hypoxia-induced PAH (38) (**Figure 1**).

### 2.3 Pyroptosis

PCD encompasses various forms of cell death, namely, apoptosis, autophagy, and pyroptosis, which are regulated by unique host proteins. In contrast to apoptosis, pyroptosis is a necrotic and inflammatory programmed cell death induced by inflammasome-associated caspases, such as caspase 1, caspase 4, caspase 5, and caspase 11 (mouse), whereas some apoptotic caspases, such as caspase 3 and caspase 8, also play a role in the occurrence of pyroptosis (39). Pyroptosis can be initiated through two main pathways: the typical inflammasome activation pathway (caspase 1-dependent pathway) and the atypical inflammasome activation pathway (caspase 1-independent pathway) (**Figure 1**). Traditionally, apoptosis-related caspases, such as caspase 3 and caspase 8, were not associated with pyroptosis. However, recent studies have unveiled that caspase 3 can catalyze the cleavage of GSDME, leading to the production of N-GSDME termini and consequent pyroptosis in tumor cells. In addition, caspase 8 has been found to promote the cleavage of GSDMD in mouse macrophages, which further enhances our comprehension of pyroptosis (40).

Pyroptosis may act as a crucial part of the pathogenesis of hypoxia-induced PAH, thus offering insights into potential therapeutic strategies. In the study of Wu et al., caspase 4/11 plays a key role in regulating pulmonary vascular dysfunction and accelerating the progression of PAH (41). Studies from Hu et al. demonstrated disulfiram (DSF) attenuated vascular remodeling and hypoxia-induced PAH by inhibiting GSDMD cleavage and pyroptosis in human pulmonary artery smooth muscle cells (hPASMCs) (42). Additionally, Zhang et al. found that signal transducer and activator of transcription 1 (STAT1) promoted programmed death-ligand 1 (PD-L1) upregulation and activation of caspase 1-dependent pyroptosis, thereby accelerating the progression of PAH (43). Along similar lines, He et al. identified that GLI1 affected the progression of PAH by promoting PASMC pyroptosis through the apoptosis-associated speck-like protein containing a caspase recruitment domain (ASC) pathway (44). Furthermore, a separate study revealed that G-protein coupled receptor 146 (GPR146) induced PAEC pyroptosis through the NLRP3/caspase 1 signaling axis, resulting in the promotion of endothelial injury and PAH progression (45). Wu et al. also demonstrated that KIF23 regulated the expression of caspase 3, NLRP3, and HMGB1 by inhibiting the pyroptosis and proliferation of PASMCs (46) (**Figure 1**).

### 2.4 Ferroptosis

Ferroptosis is an intracellular iron-dependent form of cell death that is distinct from apoptosis, autophagy, and pyroptosis. The characteristic morphological features of ferroptosis are mitochondrial changes, including reduction or disappearance of mitochondrial cristae, rupture of the mitochondrial outer membrane, and concentration of mitochondrial membrane (47). The process of ferroptosis is closely related to the System Xc-/GPX4 signaling pathway, iron homeostasis, and lipid oxidative metabolism (48).

Accumulating evidence supports the hypothesis that ferroptosis is involved in the progression of lung diseases. However, only a few studies have investigated the role of ferroptosis in PAH. In a recent systematic investigation conducted by Zhang et al., they revealed a substantial upregulation in the expression of all ferroptosis-associated genes in individuals with PAH. In addition, all 10 ferroptosis-associated genes exhibited positively correlated expression patterns, suggesting that PAH initiated ferroptosis (49). Another study discovered that peroxiredoxin 6 (PRDX6) facilitates ferroptosis in PAECs and instigates pulmonary vascular remodeling. This process is mediated by the release of HMGB1 and subsequent activation of the TLR4/NLRP3 pathway, thereby leading to the pathogenesis of PAH (50). Xie et al. indicated that PAEC ferroptosis stimulates the NLRP3 inflammatory response *via* the HMGB1/TLR4 pathway and participated in the progression of PAH (51). However, Hu et al. demonstrated that SLC7A11 inhibits ferroptosis and promoted proliferation in PAH, thus restoring the balance between cell death and proliferation in PASMCs (52). In fact, further studies on the role of ferroptosis in PAH are still required (**Figure 1**).

## 3 Epigenetic regulation in programmed cell death in PAH

### 3.1 RNA methylation

#### 3.1.1 Overview of RNA methylation

Epigenetics is the study that modulates heritable gene expression without DNA sequence changes, including DNA and RNA methylation, histone modification, and non-coding RNA regulation (53). m^6^A methylation is the most prevalent epigenetic modification of RNA nucleotides. Moreover, m^6^A methylation modification plays a crucial role in governing the process of RNA splicing, gene expression, transcription, translation, and nuclear export. The modification of m^6^A is reversible and mediated by “writers”, “erasers”, and “readers” (54).

**Figure 2:**
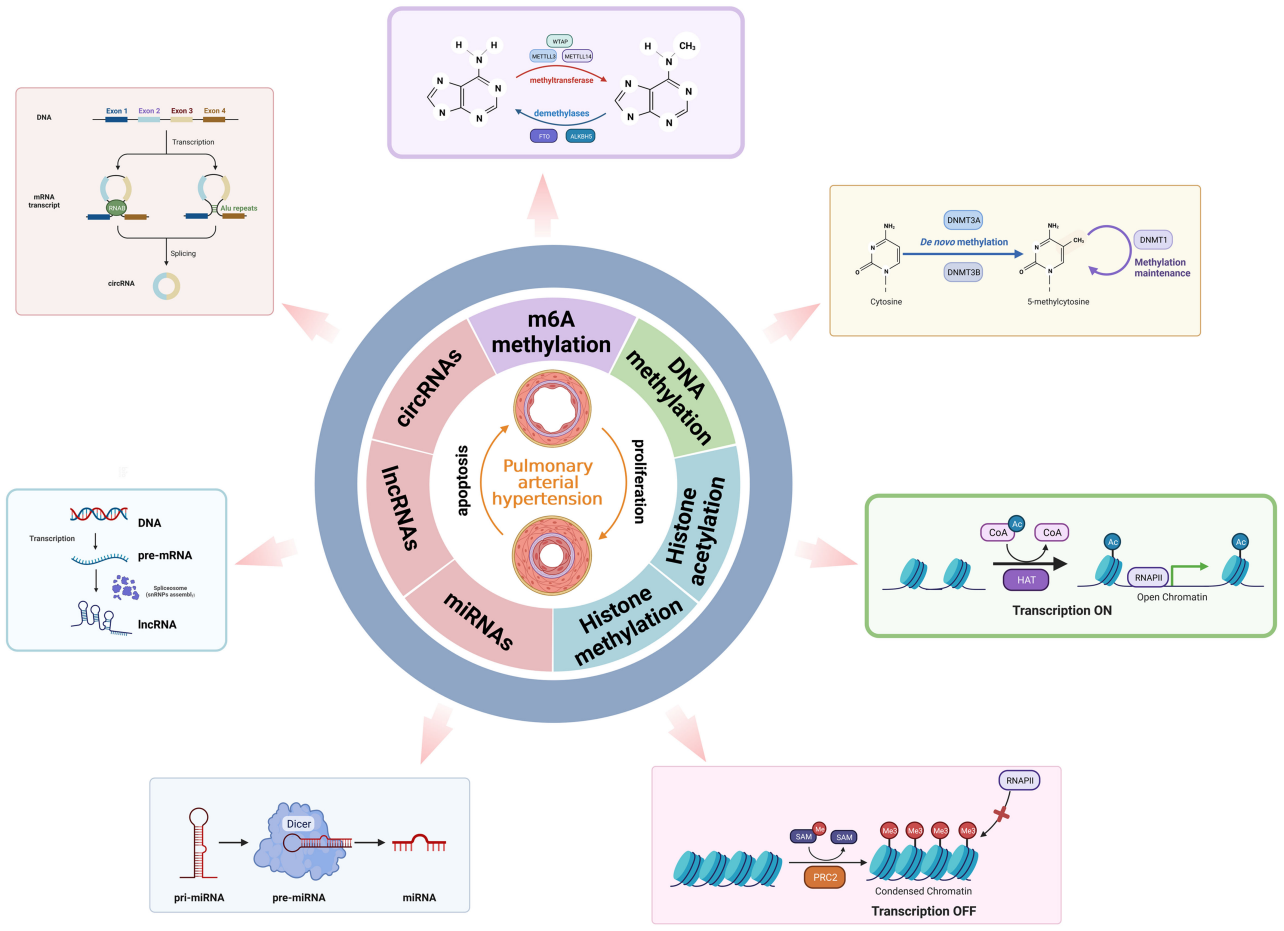
An overview of epigenetic remodeling.

The m^6^A process is mainly catalyzed by the m^6^A methyltransferase complex, including methyltransferase like 3 (METTL3), METTL14, Wilms’ tumor 1-associated protein (WTAP), RNA-binding motif protein 15 (RBM15), zinc finger CCCH-type containing 13 (ZC3H13), and KIAA1429 (55). The demethylases act as erasers in RNA molecules to remove the m^6^A modifications. RNA demethylases mainly consist of fat mass and obesity-associated protein (FTO) and alpha-ketoglutarate-dependent homolog 5 (ALKBH5). Reader proteins play a crucial role in recognizing m^6^A binding sites and interacting with them, each performing specific m^6^A-dependent biological functions (56). The m^6^A reader proteins containing the YTH domain include YTHDF1-3 and YTHDC1-2. YTHDF1 promotes mRNA translation initiation, while YTHDF2 promotes mRNA degradation. YTHDF3 interacts with YTHDF1 to promote mRNA translation or with YTHDF2 to enhance mRNA degradation. Furthermore, YTHDC1 facilitates pre-mRNA splicing and nuclear export of mRNA. YTHDC2, however, enhances the translation efficiency of target mRNA (57). Another kind of reader protein, IGF2BP1-3, promotes mRNA stability and translation in an m^6^A-dependent manner. In addition, the eukaryotic initiation factor 3 (eIF3) promotes mRNA translation (58). Moreover, the nuclear m^6^A reader HNRNPA2B1 is involved in promoting miRNA processing and mRNA splicing (59) (**Figure 2**).

#### 3.1.2 RNA methylation in programmed cell death in PAH

In the latest systematic study, the m^6^A methyltransferase METTL3 has been identified as a strong proponent of PAH development (60). Conversely, Xu et al. demonstrated that sustained low expression of METTL3 impacts the m^6^A level of PAH-related genes, consequently facilitating PAH development (61). Meanwhile, the m^6^A reading protein YTHDF1 promotes PAH by contributing to MAGED1 translation in an m^6^A-dependent manner (62). Additionally, another investigation discovered that YTHDF1 recognizes and promotes Forkhead box M1 (Foxm1) protein translation efficiency, thereby enhancing the hypoxic PAH (63). Emerging evidence supports that m^6^A is associated with PAH pathology; however, the effects of m^6^A on PCD in PAH have been scarcely reported. Accumulating evidence suggests that hypoxic signaling plays a fundamental and pivotal role in the pathogenesis of PAH (64). Supporting this notion, it has been observed that the upregulation of FTO effectively suppresses hypoxia/reoxygenation (H/R)-treated cardiomyocyte apoptosis (65, 66). In line with this notion, the expression of m^6^A reader YTHDF1 is significantly correlated with hypoxia-induced autophagy in patients with hepatocellular carcinoma (HCC) (67). Similarly, Lin et al. revealed a connection between METTL3-mediated m^6^A modification, sorafenib resistance, and autophagy in HCC under hypoxic conditions (68). Furthermore, separate investigations have demonstrated that hypoxia leads to the suppression of METTL14, resulting in enhanced SLC7A11 mRNA degradation in an m^6^A-dependent manner, which may serve as a potential therapeutic target for the ferroptosis of hepatocellular carcinoma (69). In the meanwhile, Yang et al. found that hypoxia induces long non-coding RNA (lncRNA)–CBSLR to recruit YTHDF2 protein and destabilizes CBS, and mRNA destabilizes through m^6^A-YTHDF2-dependent modulation. This process ultimately contributes to ferroptosis resistance in gastric cancer (70). Based on the diverse regulatory roles of m^6^A in hypoxic diseases, it is plausible that the m^6^A epigenetic modifications regulate signaling pathways and targets associated with programmed cell death, thereby contributing to the occurrence of PAH. Consistent with the above reports, YTHDC1-mediated m^6^A modification induces lncRNA FENDRR degradation, which subsequently promotes hypoxia-induced PAH by regulating DNA methylation of the promoter region of dynamin-related protein 1 (DRP1) (71).

### 3.2 DNA methylation

#### 3.2.1 Overview of DNA methylation

DNA methylation is one of the major epigenetic modifications in cells, which is based on the transfer of a methyl group (CH_3_^−^) from an *S*-adenosylmethionine donor to the C-5 position of a cytosine ring of DNA to form 5-methylcytosine (5-mC) (72). DNA methylation is catalyzed by three DNA methyltransferases (DNMTs): DNMT1, DNMT3A, and DNMT3B (73). Of these, DNMT1 is able to copy CpG methylation patterns and add to the newly synthesized DNA strand, which plays a role in maintaining DNA methylation status during DNA replication. Conversely, DNMT3A and DNMT3B are categorized as *de novo* methyltransferases that reversibly methylate the unmethylated CpG dinucleotides and set the initial pattern of the methyl groups on the DNA sequence (74). DNMT3-like protein, also known as DNMT3-L, is the third member of the DNMT3 family, which can increase the DNA methylation of the whole genome by activating DNMT3A and DNMT3B, thereby affecting the transcription expression of related downstream genes (75). The DNA demethylation process is performed by TET family enzymes (TET1, TET2, and TET3), which oxidize 5-methylcytosines to 5-hydroxymethylcytosines and reverse the modification (76) (**Figure 2**).

#### 3.2.2 DNA methylation in programmed cell death in PAH

Several studies have investigated that DNA methylation is associated with the vascular pathology of PAH. Specifically, studies have shown that 5-Aza-2′-deoxycytidine (5-Aza-dC), a DNA methyltransferase inhibitor, attenuates hypoxic PAH *via* demethylation of the PTEN promoter (77). Along this line, DNMT3B has been confirmed to be upregulated in both PAH patients and rat models. Overexpressing of DNMT3B in PASMCs has been shown to ameliorate hypoxia-mediated PAH (78). While the function of DNA methylation in PAH is well characterized, its understanding of the function in programmed cell death and the underlying functional machinery in PAH remain unexplored. Dysregulation of oxygen-sensing mechanisms is a common feature of both PAH and cancer, especially apoptosis resistance. Many of these abnormalities are regulated by epigenetic modifications (79). Therefore, we hypothesize that the DNA methylation mechanism in programmed cell death in PAH under hypoxic conditions may be similar to that in cancer. Supporting this hypothesis, Mamo et al. demonstrated that the demethylation of intron 18 of epidermal growth factor receptor (EGFR) restored the hypoxic regulation of EGFR, leading to apoptosis resistance and migration (80). In the study of Feng et al., it was found that hypermethylated gene ankyrin repeat and death domain-containing 1A (ANKDD1A) is a tumor suppressor in glioblastoma multiforme (GBM). The recovery of ANKDD1A expression results in reduced transactivation function and stability of HIF1α to inhibit autophagy and induce apoptosis in the hypoxic microenvironment (81). Recent research indicates that DNA methylation modifier lymphoid-specific helicase (LSH) interacts with WDR76 to impede ferroptosis. However, EGLN1 and c-Myc directly activate the expression of LSH by inhibiting HIF-1α (82). Due to the regulatory role in tumors, DNA methylation may regulate targets associated with programmed cell death in PAH. Indeed, DNA methylation in the promoter region of BMPR2 induces PAH by regulating BMP signaling pathways and increasing cell apoptosis (83). It is worth noting that more investigations are required to ascertain the involvement of DNA methylation in the processes of ferroptosis, autophagy, and pyroptosis in PAH.

### 3.3 Histone modification

#### 3.3.1 Overview of histone modification

In eukaryotic cells, the nucleosome is the basic unit of chromatin, which is comprised of a histone octamer with one H2A–H2B tetramer and two H3–H4 dimers surrounded by 146–147 base pairs of double helix DNA (84). The N-terminal and C-terminal tails of histone can be modified for post-translational modification catalyzed by enzymes, directly affecting chromatin status and gene expression. Histone modification includes histone acetylation, histone methylation, and other modifications such as histone phosphorylation and histone ubiquitination (85). Acetylation of lysine residues reduces the positive charge, hindering the interaction between histone tails and negatively charged DNA. Consequently, chromatin structure is relaxed, enabling exposure of underlying DNA and facilitating transcriptional activation (86). Histone acetylation at lysine residues is catalyzed by histone acetyltransferases (HATs) to induce transcriptional activation. Histone deacetylation is regulated by histone deacetylases (HDACs), leading to transcriptional inhibition (87). Histone methylation is an extensively researched post-translational modification of histones. Histone methylation usually occurs at the arginine, lysine, and histidine residues of histone H1, H2A, H2B, H3, and H4 by adding methyl groups. The arginine residue methylation can be mono-(me) and di-(me2) methylated, while lysine residues can be mono-(me), di-(me2), and tri-(me3) methylated (88). The process of histone methylation is catalyzed by the histone methyltransferase (HMT), which transfers methyl groups to lysine, arginine, or histidine residues of histones by using *S*-adenosine methionine (SAM). Additionally, most histone modifications are reversible. Methyl groups are removed from lysine, arginine, or histidine residues by histone demethylases (HDMs) (89). Lysine-specific demethylase 1 (LSD1) is the first demethylase to remove the methylation at H3K4 and H3K9. Histone phosphorylation occurs on residues of serine, threonine, and tyrosine. Histone phosphorylation and histone dephosphorylation are regulated by protein kinase (PK) and protein phosphatase (PP) in a state of homeostasis (90). The methylation sites H3K9 and H3K27 share the same serine residue and can be phosphorylated. Based on the different histones and modification sites, histone phosphorylation is associated with chromosome condensation, gene transcription, and the DNA damage repair process. Furthermore, other modifications such as histone ubiquitination and histone ADP-ribosylation regulate gene transcription in various directions (91) (**Figure 2**).

#### 3.3.2 Histone modification in programmed cell death in PAH

Recently, an expanding body of evidence has highlighted that histone modification is a promising strategy for the treatment of PAH. In a systematic study, Qi et al. reveal a pivotal role of the histone modifier SUV4-20H1. Inactivation of Suv4-20h1 increased expression of the secreted superoxide dismutase 3 (Sod3), resulting in an imbalance of reactive oxygen species (ROS) in the alveolar and pulmonary vascular ventricles, ultimately leading to PAH (92). Bisserier et al. found that SIN3a regulates BMPR2 expression and pulmonary vascular remodeling by a dual mechanism. On the one hand, SIN3a inhibits EZH2 expression and decreases the levels of H3K27me3 in the promoter region of BMPR2. On the other hand, the methylation level of the BMPR2 promoter is decreased by upregulating TET1 and inhibiting DNMT1 activity (93). However, it remains unclear whether histone modification regulates PAH by targeting PCD. Shedding light on this aspect, studies have demonstrated that the acetylation of vestigial-like family member 4 (VGLL4) inhibits PASMC apoptosis and pulmonary arterial remodeling through signal transducer and activator of transcription 3 (STAT3) signaling (94). Moreover, RVX208, a clinically available BET inhibitor, has the potential to modulate anti-apoptotic and proinflammatory pathways through interactions with FoxM1 and PLK1. This discovery supports the establishment of a clinical trial of RVX208 in patients with PAH (95). Another study has revealed the detrimental effects of HDAC inhibitor trichostatin A (TSA) on RV remodeling under pressure overload may be achieved through antiangiogenic or proapoptotic effects (96). Based on these findings, histone modification is significant in PASMC apoptosis during the PAH process; however, knowledge of the contribution of histone modification in regulating other types of PCD in PAH remains fairly limited so far.

### 3.4 Non-coding RNA molecules

Non-coding RNAs (ncRNAs) can be divided into two types based on their length: small ncRNAs (sncRNAs), which consist of fewer than 200 nucleotides, including microRNAs (miRNAs), and lncRNAs, which are longer than 200 nucleotides (97). The three major types of ncRNAs (miRNAs, lncRNAs, and circRNAs) are involved in the disease onset and progression of PAH (**Figure 2**).

#### 3.4.1 MicroRNAs

MiRNAs are the most extensively studied endogenous RNAs of approximately 22 nucleotides (98). The biological functions of miRNAs depend on complementary targeting to the 3′-untranslated region (UTR) of mRNAs and then negatively regulate the expression of target genes at the post-transcriptional level (99). In a study conducted by Russomanno et al., miR-150 was shown to reduce the expression of inflammation-, apoptosis-, and fibrosis-related genes in the pathology of PAH and enhance mitochondrial metabolic potential *via* increased expression of PTEN-like mitochondrial phosphatase (PTPMT1) (100). Chen et al. found that MiD expression is epigenetically upregulated by the decreased levels of miR-34a-3p. This upregulation promoted mitotic fission, leading to pathological proliferation and resistance to apoptosis (101). In the prospective study, the secretion of miR-195-5p by anti-apoptotic endothelial cells was found to promote the proliferation and migration of PASMCs in PAH (102). Furthermore, miR-244-5p promotes apoptosis of PASMCs under hypoxia *via* DEGS1/PI3K/Akt signaling pathway (103). In addition, miR-15a-5p was shown to induce PASMC apoptosis in an animal model of PAH through the vascular endothelial growth factor (VEGF)/p38/MMP-2 signaling pathway (104). The modulation of the miR-143/145 cluster in PASMCs, as demonstrated by Deng et al., significantly altered cell migration and apoptosis (105). MiR-760, a microRNA, plays a regulatory role in hypoxia-induced hPASMC proliferation, migration, and apoptosis by targeting toll-like receptor 4 (TLR4) (106). In the study of Cai et al., miR-125a-5p ameliorates PAHs by directly targeting STAT3 to regulate PASMC proliferation and apoptosis and has a negative feedback regulation with TGF-β1 and IL-6 (107). Zhu et al. indicated that miR−371b−5p inhibits endothelial cell apoptosis in PAH *via* PTEN/PI3K/Akt signaling pathways (108). Another study further found the MFF-SIRT1/3 axis, regulated by miR-340-5p, improved mitochondrial homeostasis and proliferation–apoptosis imbalance of hypoxia-treated PAMSCs (109). Moreover, miR-874-5p was found to regulate autophagy and proliferation in PASMCs by targeting Sirtuin3 (110). In addition, miR-204 was shown to attenuate endothelial–mesenchymal transition by enhancing autophagy in hypoxia-induced PAH (111). Ou et al. reported that miR-let−7d alleviates PAH by inhibiting the autophagy of PAECs and suppressing endothelin synthesis through negative regulation of autophagy−related 16−like 1 (ATG16L1) (112). In conclusion, numerous studies have provided clear demonstrations of miRNAs with programmed cell death in hypoxia-induced PAH; however, other ncRNAs still need to be further studied in the same manner.

#### 3.4.2 Long non-coding RNAs

LncRNAs are a class of ncRNAs greater than 200 bp in length, with low expression levels and wide tissue specificity. LncRNAs have a complex regulatory mechanism in the nucleus and cytoplasm by directly binding to DNA, RNA, and proteins to regulate gene expression (113). Recently, several studies have investigated the impact of lncRNAs on the pathogenesis of PAH. For instance, one study showed that silencing of lncRNA SOX2-OT attenuates hypoxia-induced hPASMC proliferation, migration, anti-apoptosis, and inflammation by modulating the miR-455-3p/SUMO1 axis (114). In the meanwhile, Li et al. identified that lncRNA HOXA-AS3 suppresses hPASMC apoptosis *via* regulation of miR-675-3p/PDE5 axis (115). Additionally, overexpression of lncRNA Ang362 decreases apoptosis of hPASMCs by regulating miR-221 and miR-222 (116). Notably, studies have indicated that lncRNA TCONS_00034812 regulates PASMC proliferation and apoptosis and participates in vascular remodeling during PAH (117). Furthermore, lncRNA PVT1 was found to promote the mRNA and protein expression of serum response factor (Srf) and CTGF by suppressing miR-26b and miR-186, leading to deregulation of autophagy and abnormal proliferation of PASMCs (118). Another study reported that the lncRNA−GAS5/miR−382−3p axis inhibits pulmonary artery remodeling and promoted autophagy in PAH (119). Along this line, studies by Li et al. pointed to lnc-Rps4l inhibiting hypoxia-induced PASMC pyroptosis through the encoded peptide RPS4XL (120).

#### 3.4.3 Circular RNAs

Circular RNAs are a unique class of lncRNAs that are directly produced by back-spliced exons and introns, thus establishing a covalent closed-loop structure. Circular RNAs regulate gene expression through transcriptional or post-transcriptional mechanisms, such as regulating miRNA target genes, regulating RBP-dependent functions, recruiting proteins, and even producing unique peptides (121). Due to the functional diversity of circRNAs, several articles have reported that circRNAs regulate signaling pathways and targets relevant to PAH. For instance, data from Jiang et al. found that circ-Calm4 functions as a competitive endogenous RNA to regulate the expression of miR-124-3p and exacerbate hypoxia-induced PASMC pyroptosis (122). Concordant with this scenario, circ-Calm4 was also confirmed to regulate hypoxia-induced PASMC autophagy by binding Purb (123). Similarly, circ-Sirtuin1 has been shown to mitigate PAH by improving PASMC proliferation, migration, and autophagy by targeting miR-145-5p/protein kinase-B3 axis under hypoxic environments (124). Interestingly, Jin et al. analyzed circRNA profiles in whole-blood samples and found that circ-NFXL1_009 attenuates hypoxia-induced proliferation, apoptotic resistance, and migration of PASMCs (125). Furthermore, circ_0016070 has been implicated in reducing hypoxia-induced apoptosis in PAHs by interacting with miR-340-5p/TCF4/β-catenin/TWIST1 signaling pathway (126). Collectively, the prevailing mechanism of action for most circRNAs in PAH involves functioning as miRNA sponges. However, the other roles and molecular mechanisms of circRNAs have not been fully elucidated (**Table 1**).

**Table 1:** NcRNAs and their function in hypoxia-induced PAH.

NcRNAs	Expression	Functional role (PCD)	Molecular targets	References
miR-150	Down	Apoptosis	PTPMT1	100
miR-34a-3p	Down	Apoptosis	MiD	101
miR-195-5p	Up	Apoptosis	Smad7	102
miR-244-5p	Up	Apoptosis	DEGS1	103
miR-15a-5p	Up	Apoptosis	VEGF/p38/MMP−2	104
miR-143/145	Up	Apoptosis	–	105
miR-760	Down	Apoptosis	TLR4	106
miR-125a-5p	Down	Apoptosis	STAT3	107
miR−371b−5p	Down	Apoptosis	PTEN/pI3K/Akt	108
miR-340-5p	Down	Apoptosis	IL-1β and IL-6	109
miR-874-5p	Up	Autophagy	Sirt 3	110
miR-204	Down	Autophagy	ATG7	111
miR-let−7d	Down	Autophagy	ATG16L1	112
LncRNA SOX2-OT	Up	Apoptosis	miR-455-3p	114
LncRNA HOXA-AS3	Up	Apoptosis	miR−675−3p	115
LncRNA Ang362	Up	Apoptosis	miR-221/miR-222	116
LncRNA TCONS_00034812	Down	Apoptosis	Stox1	117
LncRNA PVT1	Up	Autophagy	miR-26b/miR-186	118
LncRNA GAS5	Down	Autophagy	miR−382−3p	119
LncRNA Rps4l	Up	Pyroptosis	ILF3	120
Circ-Calm4	Up	Pyroptosis	miR-124-3p	122
Circ-Calm4	Up	Autophagy	Purb	123
Circ-SIRT1	Up	Autophagy	miR-145-5p	124
Circ-NFXL1_009	Down	Apoptosis	hsa-miR-29b-2-5p	125
Circ-0016070	Up	Autophagy	miR-340-5p	125

ncRNAs, non-coding RNAs; PAH, pulmonary arterial hypertension; PCD, programmed cell death; VEGF, vascular endothelial growth factor.

## 4 Conclusion and prospects

PAH is a complex progressive disease, which involves multiple cellular processes. The hyperproliferation and anti-apoptosis of PASMCs are the basic pathophysiological processes of PAH. Based on the studies presented in our review, other forms of programmed cell death, such as autophagy, pyroptosis, and ferroptosis, have been shown to be involved in the development of PAH. Therefore, a better understanding of the processes and mechanisms of programmed cell death involved in PAH will provide novel therapeutic strategies. Research studies have found that epigenetic modification plays a crucial role in the pathological process of PAH; therefore, exploring the epigenetic modification of PAH may be a new treatment strategy (127). Epigenetic modifications are involved in programmed cell death processes at different levels. Multiple lines of evidence indicate that epigenetic alterations, including regulation mediated by ncRNAs, play a significant role in apoptosis, autophagy, and pyroptosis in PAH (128). Although current evidence provides epigenetic modifications that regulate signaling pathways associated with programmed cell death, a significant proportion of research studies have focused on ncRNAs. Other epigenetic modifications such as methylation and acetylation as well as phosphorylation should be further studied, as they may be important contributors to the pathogenesis of PAH. In particular, there is substantial evidence that HDAC inhibitors may be effective anti-cancer agents, especially when used in combination with conventional chemotherapy drugs. As such, regulating these HDACs may also have therapeutic potential for PAH (129). Despite this progress, the relationship between histone modification and programmed cell death in hypoxia-induced PAH remains largely unexplored. Furthermore, DNA methylation has been associated with gene silencing and has been shown to regulate apoptosis in the pathogenesis of PAH (11). However, our current understanding of this intricate process is still very limited. In addition, direct evidence on other DNA methylation-mediated types of programmed cell death in PAH remains lacking. Therefore, more studies are still needed to reveal the complex mechanisms of connecting epigenetic modification factors and different modes of programmed cell death during hypoxia-induced PAH.

## Author contributions

YJ, SS, and JXL designed and wrote the manuscript. XG and JYL collected documents. CY, QF, and BZ revised and edited the manuscript. All authors read and approved the final version of the manuscript.
